# Spatial Scattering Radiation to the Radiological Technologist during Medical Mobile Radiography

**DOI:** 10.3390/bioengineering10020259

**Published:** 2023-02-16

**Authors:** Kazuki Otomo, Yohei Inaba, Keisuke Abe, Mana Onodera, Tomohiro Suzuki, Masahiro Sota, Yoshihiro Haga, Masatoshi Suzuki, Masayuki Zuguchi, Koichi Chida

**Affiliations:** 1 Course of Radiological Technology, Health Sciences, Tohoku University Graduate School of Medicine, 2-1 Seiryo, Aoba-ku, Sendai 980-8575, Japan; 2 Department of Radiology, Tohoku University Hospital, 1-1 Seiryo, Aoba-ku, Sendai 980-8574, Japan; 3 Department of Radiation Disaster Medicine, International Research Institute of Disaster Science, Tohoku University, 468-1 Aramaki Aza-Aoba, Aoba-ku, Sendai 980-0845, Japan; 4 Department of Radiology, Sendai Kousei Hospital, 4-5 Hirose-machi, Aoba-ku, Sendai, 980-0873, Japan

**Keywords:** radiation protection and safety, mobile radiography, radiological technologist, scatter radiation, eye lens dose, disaster medicine, occupational radiation exposure, X-ray examination, medical radiation dose, radiation dose limit

## Abstract

Mobile radiography allows for the diagnostic imaging of patients who cannot move to the X-ray examination room. Therefore, mobile X-ray equipment is useful for patients who have difficulty with movement. However, staff are exposed to scattered radiation from the patient, and they can receive potentially harmful radiation doses during radiography. We estimated occupational exposure during mobile radiography using phantom measurements. Scattered radiation distribution during mobile radiography was investigated using a radiation survey meter. The efficacy of radiation-reducing methods for mobile radiography was also evaluated. The dose decreased as the distance from the X-ray center increased. When the distance was more than 150 cm, the dose decreased to less than 1 μSv. It is extremely important for radiological technologists (RTs) to maintain a sufficient distance from the patient to reduce radiation exposure. The spatial dose at eye-lens height increases when the bed height is high, and when the RT is short in stature and abdominal imaging is performed. Maintaining sufficient distance from the patient is also particularly effective in limiting radiation exposure of the eye lens. Our results suggest that the doses of radiation received by staff during mobile radiography are not significant when appropriate radiation protection is used. To reduce exposure, it is important to maintain a sufficient distance from the patient. Therefore, RTs should bear this is mind during mobile radiography.

## 1. Introduction

In radiation medicine, the patient radiation doses [1,2,3,4] and occupational exposure [5,6,7,8] are important problems [9,10,11,12]. Radiology presents a risk of radiation-induced patient injuries, such as skin erythema, particularly in interventional radiology [13,14,15,16]. Likewise, in radiology workers, there is also the risk of radiation-induced injuries, such as cataracts [17,18,19,20,21].

Mobile radiography makes it possible to examine patients who have difficulty moving and is performed in various locations, such as general wards, intensive care units (ICUs), and operating rooms [22,23,24]. In such contexts, there is sometimes little distance between the radiological technologist (RT) and the patient because the RT must accurately confirm the patient’s condition (e.g., breathing status) during X-ray procedures. Thus, exposure assessment and radiation protection for RTs are important.

By definition, mobile radiography systems are portable and have applications in wards, ICUs, operating theaters, and homes [25,26].

In the event of another pandemic such as COVID-19, mobile radiography will be in high demand because it reduces the risk of infectious spread and the need for disinfection when moving patients [27,28]. Yeung et al. reported that the use of mobile X-ray devices during the COVID-19 pandemic increased by approximately 1.7 fold [29]. Overall, mobile radiography is becoming increasingly important.

Currently, the International Commission on Radiological Protection (ICRP) recommends an occupational equivalent dose limit of 20 mSv/year for the eye over a 5-year period, with no more than 50 mSv being delivered in any single year [30]. This is significantly lower than the previous limit of 150 mSv/year, reflecting a greater acknowledgment of the importance of dose assessment and radiation protection for the eye lens [31,32,33,34,35,36,37,38].

Occupational exposure during mobile radiography is mostly due to scattered radiation from patients. The spatial scattering radiation dose is reduced to the background radiation level at a distance of about 2 m from the patient [39]. However, it is not always possible to maintain such a distance during mobile radiography. Therefore, eye-lens protection is important during this procedure.

The Monte Carlo method can simulate spatial scattering during radiography [40]. Several recent studies used Monte Carlo methods to simulate spatial scattering during interventional and mobile radiography [41,42,43]. In particular, Monte Carlo platforms, such as MCNP, GATE, and EGS, are among the gold standards for radiation dosimetry and radiation transport [44,45,46]. However, the results are only predictions and may differ from real values. Thus, it is important to measure scattered radiation using a phantom.

Although several studies have investigated radiation exposure during radiography [39,47,48], there have been few detailed assessments of the spatial dose to the eye lens. Evaluation of occupational eye radiation doses to RTs is important, and RT eye exposure during mobile radiography procedures remains unclear. In addition, bed height tends to be high in ICUs, and no reports have measured the spatial dose according to bed height. Evaluation using spatial-distribution mapping of scattered radiation is also useful for the evaluation of occupational radiation doses and protection therefrom. Therefore, we conducted a phantom study to measure the spatial dose during mobile radiography and discussed appropriate radiation-protection methods.

## 2. Materials and Methods

### 2.1. Experimental Setup

**Figure 1:**
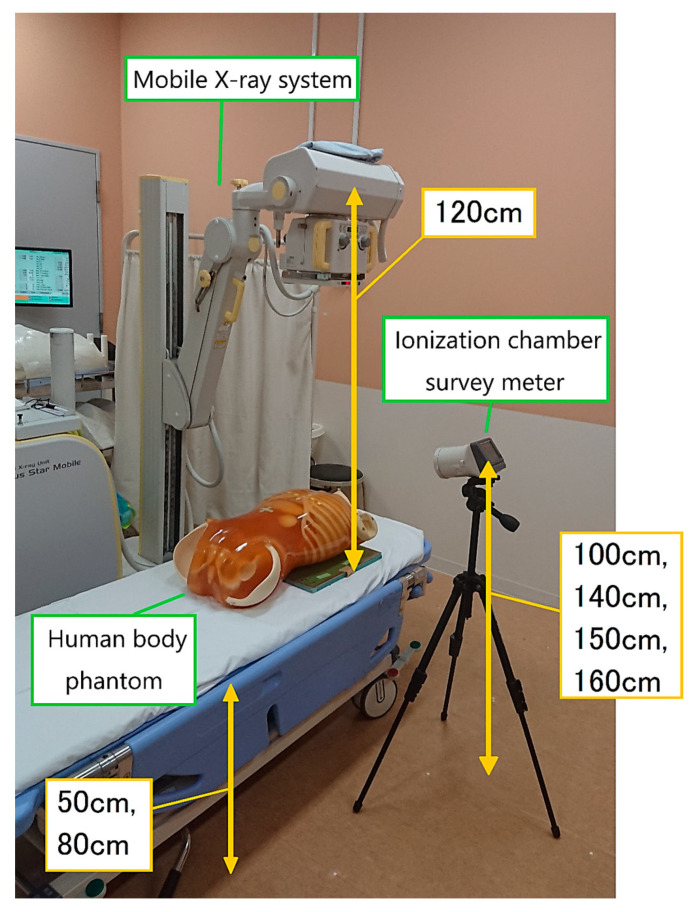
Experimental setup. The measurement points of 140 and 160 cm roughly correspond to the position of the eye lens of the radiological technologist.

Figure 1 shows the measurement setup. An inverter-type mobile X-ray system (Sirius Star Mobile; Hitachi, Tokyo, Japan) was used. A trunk phantom (PBU-60; Kyoto Kagaku Co., Ltd., Kyoto, Japan) was used to simulate a patient. We used an ionization chamber for the real-time measurement of scattered radiation. The spatial scattering radiation (1 cm dose equivalent, µSv) was measured using an ionization chamber survey meter (ICS-323C; Hitachi Aloka Medical, Ltd., Tokyo, Japan; photon energy range, 30 keV~1.5 MeV). Radiation dose calibration was performed by Hitachi Aloka Co., Ltd., Tokyo, Japan, based on the national standard. A computed radiography (CR) cassette loaded with an imaging plate (Fujifilm Corporation, Tokyo, Japan) was placed on the back of the phantom. The CR cassette was 14 × 14 inches for chest radiography and 14 × 17 inches for abdominal radiography. In addition, an anti-scatter grid (grid ratio = 5:1) was placed in front of the CR cassette.

We set the source-to-image-receptor distance (SID) to 120 cm, the height of the bed to 50 or 80 cm, and the height of the measurement points to 100, 140, 150, and 160 cm.

### 2.2. Measurement Points

**Figure 2:**
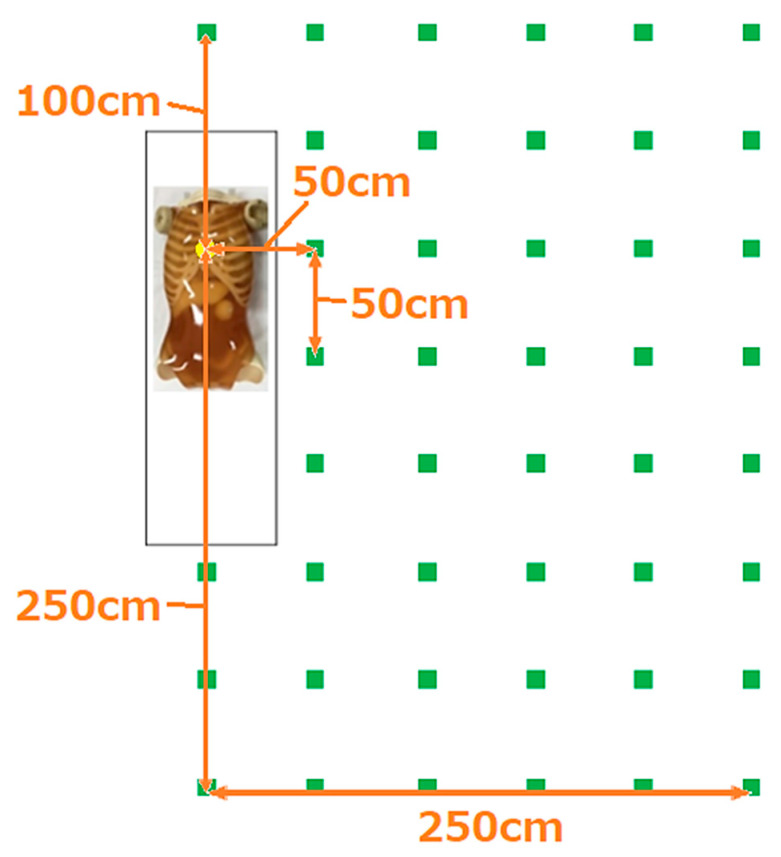
Measurement points. The measurement points (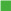) were separated by a distance of 50 cm. We measured the scattered dose during mobile radiography according to the bed and measurement point heights.

The measurement points are shown in Figure 2. The distance between each measurement point was 50 cm. Measurements were made at a total of 44 points up to 250 cm laterally, 100 cm cranially, and 250 cm caudally from the center of the X-ray. At measurement heights of 140 and 160 cm, measurements were taken at 28 points up to 150 cm laterally from the center of the X-ray. In an experiment assessing the effects of X-ray field size, measurements were taken at 14 points up to 100 cm laterally and 100 cm caudally from the center of the X-ray. Three measurements were taken at each point and the average values were obtained. No RT phantom was used during the measurements; therefore, radiation scattered from the RT was neglected. A distribution map of the spatial dose was created using SS-3030 software (SS Techno, Nagoya, Japan). This software yields two-dimensional distribution maps, rather than three-dimensional plots of volume exposure.

All figures (b) show means ± standard deviations. For all figures (b) except Figure 8b, curves were fitted using power approximation.

### 2.3. X-ray Conditions

The X-ray conditions were in accordance with those typically used in the facility where the measurements were performed (chest imaging: tube voltage, 94 kVp; tube current-time product, 4 mAs; abdominal imaging; tube voltage, 84 kVp, tube current-time product, 16 mAs).

The measurable range of the ionization chamber survey meter was 0.0–9.9 μSv. In the abdominal examination (16 mAs), when the dose exceeded the upper limit of the survey meter, the measurement was performed at 8 or 4 mAs (conversion: the measurement value × 2 when 8 mAs, the measurement value × 4 when 4 mAs).

### 2.4. Radiation Measurements

ICU beds are often set off high from the floor to facilitate medical treatment for medical staff. We compared spatial doses between bed heights of 50 cm (typical for a general ward) and 80 cm (typical for an ICU). We compared spatial doses among measurement heights of 100, 140, 150, and 160 cm. The measurement heights of 140, 150, and 160 cm roughly correspond to the eye lens of the RT.

As the X-ray conditions are different between chest and abdominal imaging, the spatial dose was also expected to differ. Therefore, we compared the spatial doses for these two areas.

For chest radiography, we also measured the scattered radiation dose for X-ray field sizes of 14 × 14 and 14 × 17 inches.

## 3. Results

In this phantom study, we assessed the scattered radiation exposure of RTs during mobile radiography. In many instances, the three measurements were very similar, although uncertainties naturally existed. Consequently, the standard deviations were zero or near-zero.

**Figure 3:**
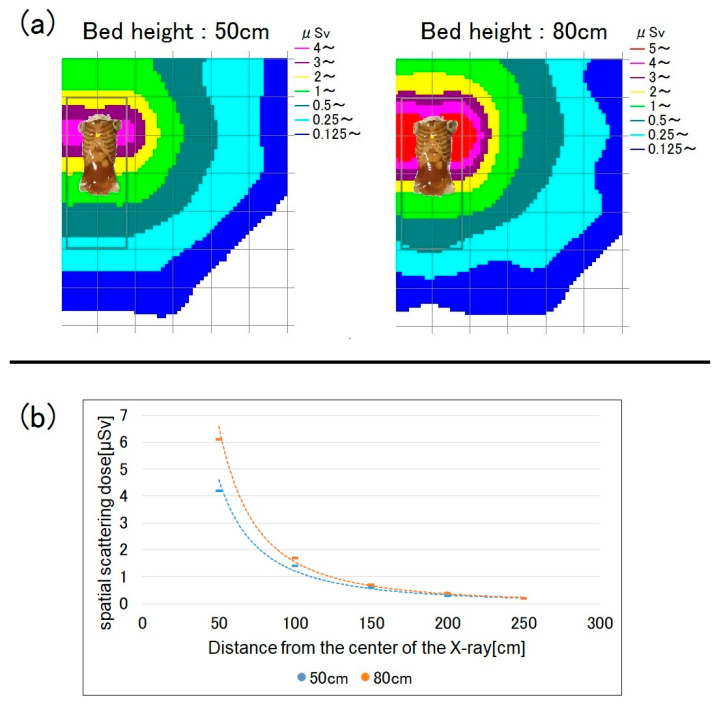
Spatial scattered radiation dose by bed height (50 vs. 80 cm) at a measurement height of 150 cm. (**a**) Dose-distribution map and (**b**) graph showing the spatial doses. When the bed height was 80 cm, the scattered dose was higher, although the difference became smaller as the distance from the X-ray center increased.

**Figure 4:**
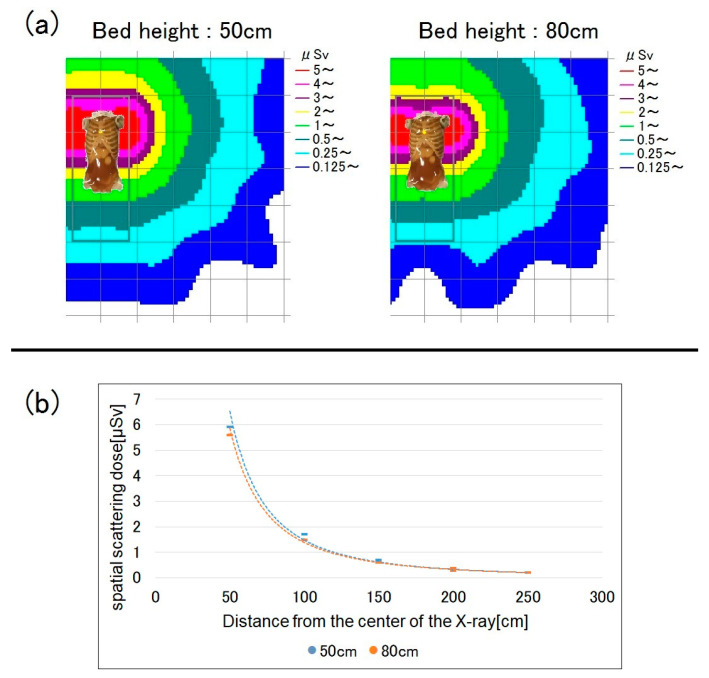
Spatial scattered radiation dose by bed height at a measurement height of 100 cm. (**a**) Dose-distribution map and (**b**) graph showing the spatial doses.

When the bed is higher, the human phantom (i.e., the source of scattered radiation) is closer to the ionization chamber survey meter. Accordingly, the scattered radiation dose increases. Figure 3 shows a map of the dose distribution by bed height and a graph of the spatial dose at a measurement height of 150 cm (i.e., roughly at eye lens level). At a bed height of 80 cm, the spatial dose increased by up to 45% compared to when it was 50 cm, while the dose at the measurement height of 100 cm showed little difference from that at 80 cm (Figure 4). As the distance from the center of the X-ray increased, the spatial dose significantly decreased, and the difference according to bed height also decreased. At a distance of 150 cm or more, there were almost no differences in spatial dose according to bed height.

**Figure 5:**
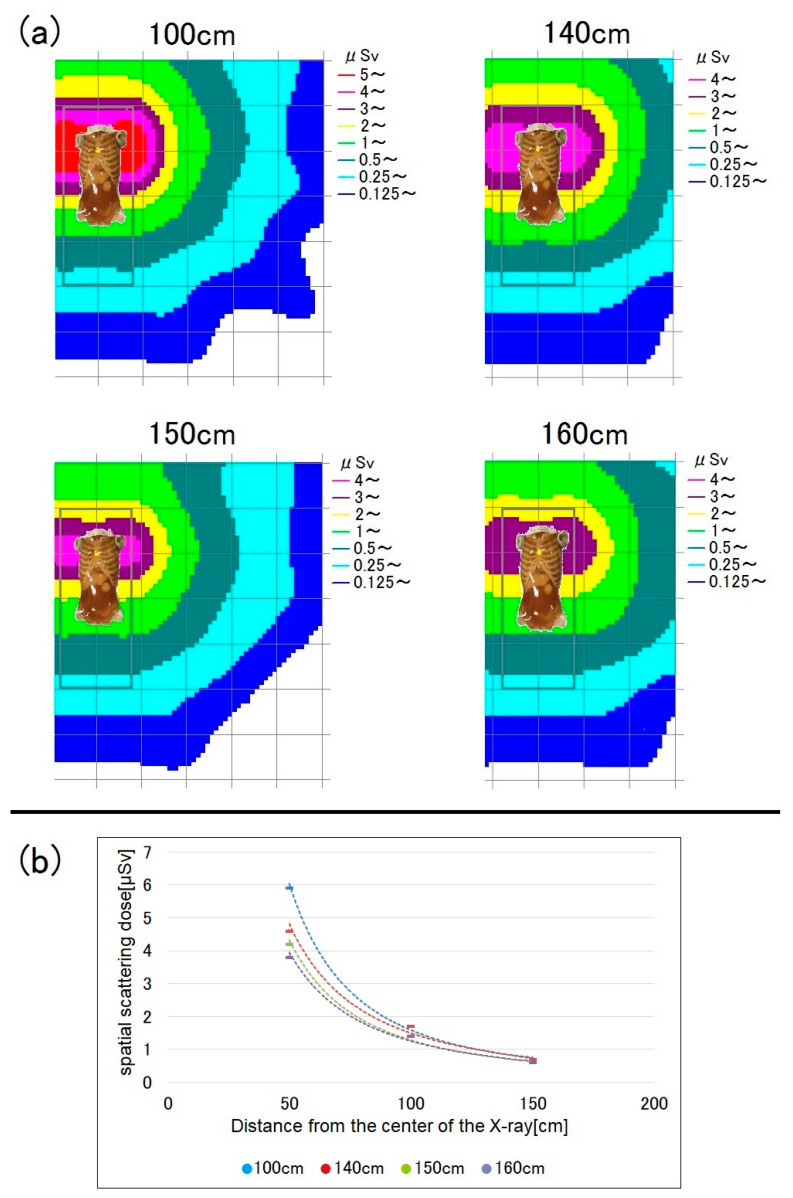
Spatial scattered radiation dose by measurement height (100, 140, 150, and 160 cm) at a bed height of 50 cm. Lower measurement points were associated with higher spatial doses. (**a**) Dose-distribution map and (**b**) graph showing the spatial doses.

**Figure 6:**
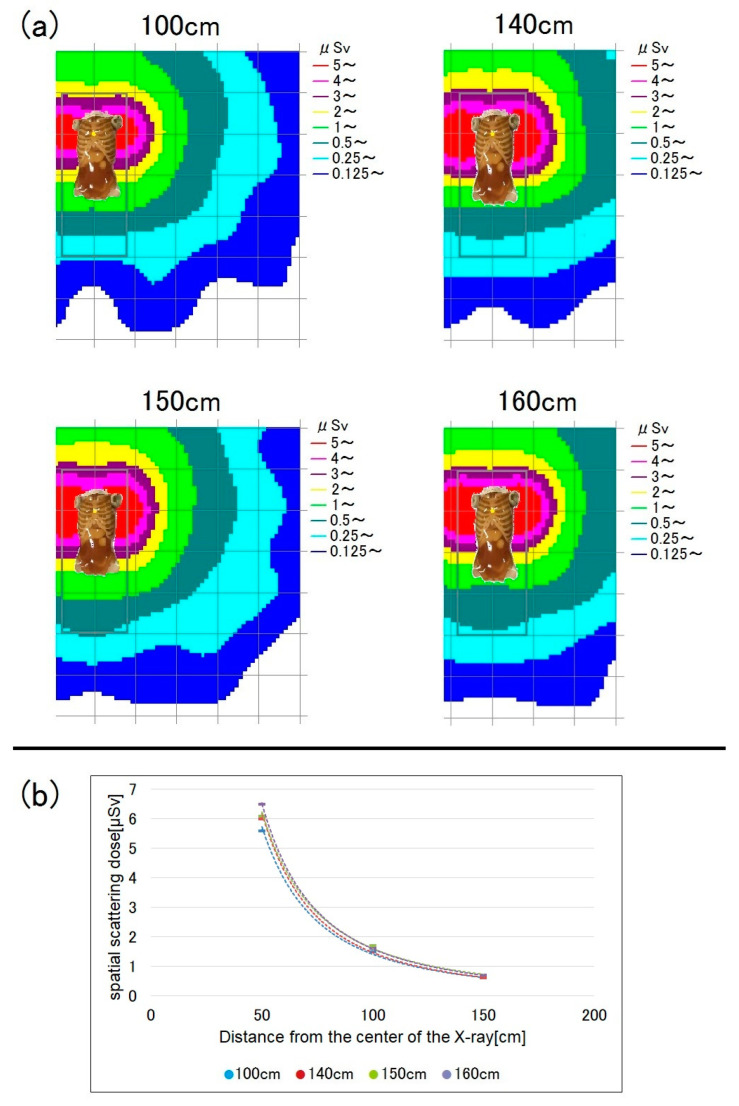
Spatial scattered radiation dose by measurement height at a bed height of 80 cm. (**a**) Dose-distribution map and (**b**) graph showing the spatial doses.

Figure 5 shows the radiation doses for chest radiography at a bed height of 50 cm. For low measurement points, the spatial dose tended to be higher. Figure 6 shows the doses for a bed height of 80 cm; at this bed height, there were no significant differences in dose between measurement points.

**Figure 7:**
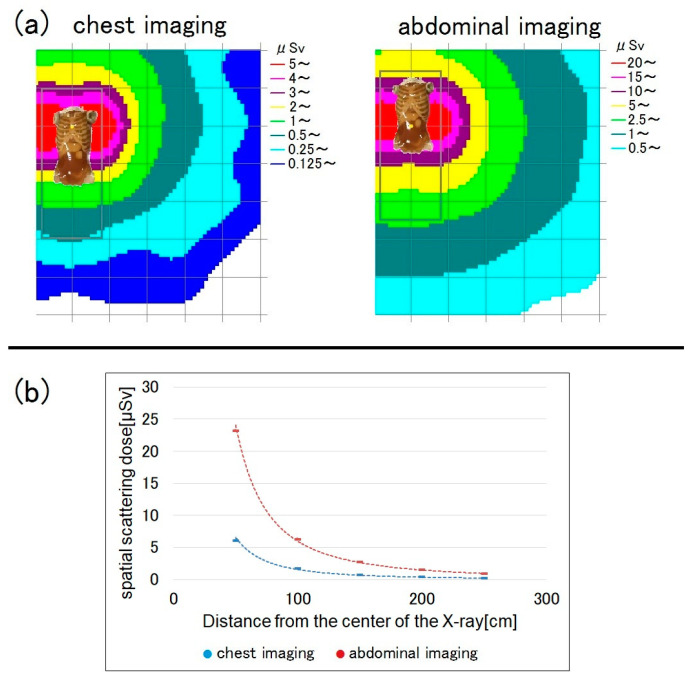
Comparison of the spatial scattered radiation dose between chest and abdominal imaging (bed height = 80 cm, measurement height = 150 cm). (**a**) Dose-distribution map and (**b**) graph showing the spatial doses.

Figure 7 shows the spatial doses for chest and abdominal imaging at a bed height of 80 cm and measurement height of 150 cm. For abdominal radiography, the dose was about three- to four-fold higher than for chest radiography.

**Figure 8:**
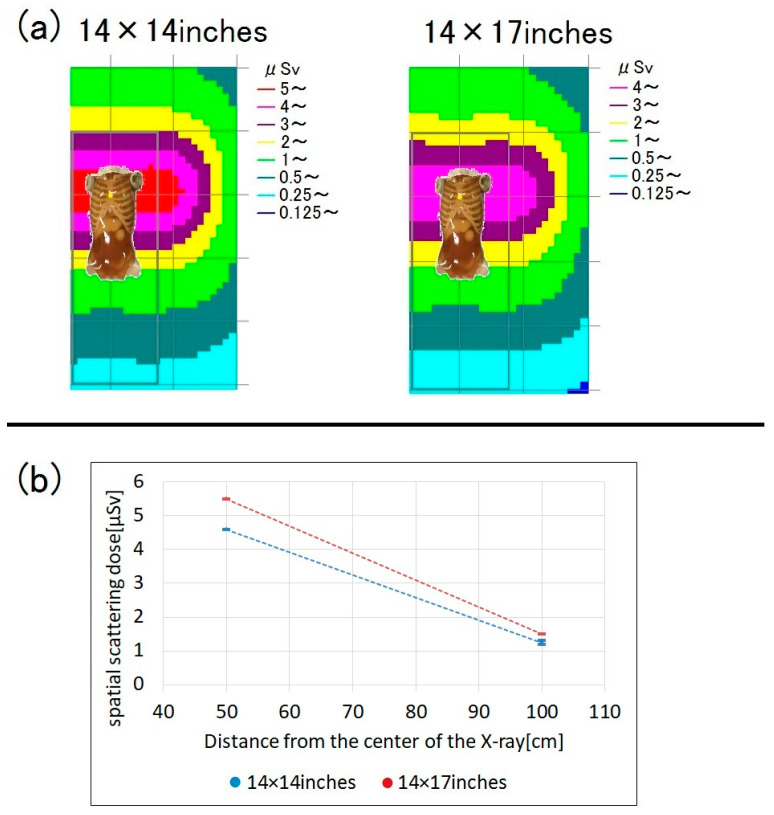
Spatial scattered radiation dose during chest radiography according to X-ray field size (14 × 14 vs. 14 × 17 inches; bed height = 80 cm; measurement height = 150 cm). (**a**) Dose-distribution map and (**b**) graph showing the spatial doses.

We compared the spatial dose between X-ray field sizes of 14 × 14 and 14 × 17 inches, with the latter dimension (17 inches) of both fields being in the cranio-caudal direction. The larger field increased the spatial dose by about 20% (Figure 8).

Table 1 summarizes our study results. There were very few uncertainties in our study data, so the standard deviations were near zero.

## 4. Discussion

In radiological examinations, it is important to evaluate/measure the exposure of patients [49,50,51] and medical staff to radiation [52,53,54,55]. Our laboratory has performed many studies on medical radiation measurement and protection [56,57,58,59,60,61,62,63]. Investigations into occupational radiation doses among RTs are very limited [64,65]. The evaluation of the occupational radiation exposure of medical radiology staff, and protection from it, are important issues [66,67,68]. At present, mobile radiography is performed in many hospitals. In portable radiography, the RT is often close to the patient so that the patient can be supported and cared for. Thus, exposure assessment and radiation protection for RTs, in particular, is important. However, the use of personal protective equipment and awareness of radiation exposure differ among facilities and individuals. Mobile radiography allows for the diagnostic imaging of patients who cannot move to the examination room. Therefore, mobile X-ray equipment is useful for patients who have difficulty with movement. However, staff are exposed to scattered radiation from the patient and can receive potentially harmful radiation doses during radiography. In addition, no detailed survey on current mobile radiography practice has been reported.

A dose-distribution map was generated to visualize the spread of scattered radiation. Few previous studies that have investigated occupational exposure during mobile radiography have analyzed the effects of bed and measurement height [39,47,48]; we addressed this in our study. We also found that, as the distance from the phantom increased, the spatial dose decreased markedly, similar to previous studies [47]. Therefore, it is very important that RTs maintain a sufficient distance from the patient to protect the eye lens during mobile radiography.

Wearing a protective apron is also effective in reducing radiation exposure [69]. Therefore, it is desirable that RTs who engage in mobile X-ray radiography wear protective aprons. However, as the eye lens cannot be protected by an apron, they should always be conscious of their distance from the patient. It is also important to wear lead glasses to protect the eye lens, particularly when the exposure dose is expected to be high, such as when performing a large number of mobile radiography procedures [32].

When the measurement height was 150 cm, the spatial dose increased by up to 45% for a bed height of 80 cm, compared to one of 50 cm (Figure 3). Therefore, the lens exposure dose increases when mobile radiography is performed on a high bed, such as in the ICU, so more attention should be paid to lens protection in such settings.

When the measurement height was 100 cm, there were almost no differences in spatial dose between bed heights of 50 and 80 cm (Figure 4), probably because of absorption by the phantom. At a measurement height of 100 cm, the phantom and ionization chamber survey meter were almost at the same height when the bed height was 80 cm. The scattered radiation mostly originates from the surface of the phantom. At the measurement point adjacent to the phantom (100 cm; bed height = 80 cm), the influence of scattered radiation on the total exposure dose was small due to absorption by the phantom. However, at a measurement height of 150 cm, the scattered radiation dose increased due to poor absorption by the phantom surface).

At a bed height of 50 cm, as is the case in a general ward sickroom, lower measurement points were associated with higher spatial doses (Figure 5). At a low measurement height of 100 cm, many scattered X-rays were detected because the distance to the phantom was short.

Among the 140, 150, and 160 cm measurement points, i.e., those roughly corresponding to the level of the eye lens, the dose was highest at 140 cm (Figure 5). Therefore, RTs with a short stature are likely to have higher eye-lens exposure doses during mobile radiography. In such cases, greater attention should be paid to radiation protection, particularly of the eye lens.

When the bed height was 80 cm, there were almost no differences in spatial dose by measurement height (Figure 6). Moreover, the doses were higher at 140, 150, and 160 cm compared to the equivalent measurement points at a bed height of 50 cm; the reason for this is that the phantom surface (i.e., the main source of scattered radiation) was nearer to the measurement points at a bed height of 80 cm.

The spatial dose for abdominal radiography was 3- to 4-fold higher than for chest radiography. This is because the mAs is higher and the X-ray field is wider for abdominal imaging. Therefore, radiation protection is even more important for RTs when performing abdominal imaging.

In chest imaging, the spatial dose was about 20% higher for the 14 × 17 inches X-ray field compared to the 14 × 14 inches field, although the X-ray output (in kV and mAs) was the same between the two fields. When the X-ray irradiation field is widened, the likelihood of repeated radiography is lower because the entire chest is more likely to be imaged; thus, the 14 × 17 inches field is often used for chest radiography. However, as the exposure dose increases with X-ray field expansion, it is important to set the X-ray irradiation field to an appropriate size to reduce the exposure dose.

We did not use a phantom RT and radiation scatter from the RT was neglected. Therefore, the radiation measurements were presumably underestimated.

In summary, mobile radiography allows for the diagnostic imaging of patients who are unable to be seen in the X-ray examination room. Therefore, mobile X-ray equipment is useful for patients who have difficulty with movement. However, staff are exposed to scattered radiation from the patient, and can receive potentially harmful radiation doses during radiography. The protection of staff is of utmost importance; therefore, we investigated the occupational radiation doses received by RTs, particularly eye doses, using phantom measurements. RTs can be located close to a patient (i.e., the source of scattered radiation) during mobile radiography. As eye doses can be significant, protective measures are essential for RTs. Protective aprons are important for protecting RTs, as is increasing the distance from the radiation source (i.e., the patient). Lead glasses may also be necessary for protecting the eyes of RTs. To reduce RT radiation exposure, RTs should remain distant from the patient if possible. However, because this distance may hinder verification of the patient’s condition, RTs sometimes work in close proximity to patients. This is a patient phantom study. In future, the data may need validation by comparison with personal RT dosimeter records. It is important to evaluate the radiation doses delivered to RTs during mobile radiography, as well as the scattered radiation distribution, to ensure adequate protection. Further comparison studies may be needed using the Monte Carlo method.

Limitation: This was a single-institution study, and multi-center evaluation is required.

## 5. Conclusions

We measured the scattered radiation dose delivered to RTs during mobile radiography and discussed radiation protection. We created a spatial scattered radiation dose-distribution map to visualize the spread of scattered radiation during mobile radiography. When the measurement height was 150 cm, the spatial dose increased by up to 45% for a bed height of 80 cm compared to 50 cm. Maintaining a sufficient distance from the patient is particularly effective in limiting radiation exposure of the eye lens. Therefore, RTs should bear this is mind during mobile radiography. The spatial dose at eye-lens height increases when the bed height is high, when the RT is short in stature, and when abdominal imaging is performed. In such cases, particular effort should be made to protect the eye lens from radiation.

To reduce exposure, it is important to maintain a sufficient distance from the patient. Therefore, RTs should bear this is mind during mobile radiography.

## Figures and Tables

**Table 1:** Summary of the phantom study.

	Distance from the Center of the X-ray				
	50 cm	100 cm	150 cm	200 cm	250 cm
Scattered radiation dose by bed height (measurement height: 150 cm)					
50 cm	5.9 ± 0	1.7 ± 0	0.7 ± 0	0.3 ± 0	0.2 ± 0
80 cm	5.6 ± 0	1.5 ± 0	0.6 ± 0	0.33 ± 0.05	0.2 ± 0
Scattered radiation dose by measurement height (bed height: 50 cm)					
100 cm	5.9 ± 0	1.7 ± 0	0.7 ± 0	0.3 ± 0	0.2 ± 0
140 cm	4.6 ± 0	1.7 ± 0	0.7 ± 0	-	-
150 cm	4.2 ± 0	1.4 ± 0	0.6 ± 0	0.3 ± 0	0.2 ± 0
160 cm	3.8 ± 0	1.4 ± 0	0.6 ± 0	-	-
Scattered radiation dose by measurement height (bed height: 80 cm)					
100 cm	5.6 ± 0	1.5 ± 0	0.6 ± 0	0.33 ± 0.05	0.2 ± 0
140 cm	6.0 ± 0	1.6 ± 0	0.6 ± 0	-	-
150 cm	6.1 ± 0	1.7 ± 0	0.7 ± 0	0.4 ± 0	0.2 ± 0
160 cm	6.5 ± 0	1.6 ± 0	0.7 ± 0	-	-
Comparison of the spatial dose between chest and abdominal imaging (bed height = 80 cm, measurement height = 150 cm)					
Chest imaging	6.1 ± 0	1.7 ± 0	0.7 ± 0	0.4 ± 0	0.2 ± 0
Abdominal imaging	23.2 ± 0	6.3 ± 0	2.7 ± 0	1.5 ± 0	0.9 ± 0

Average ± standard deviation (μSv).

## Data Availability

Not applicable.
